# Preparation, Physicochemical Characterization, and Cell Viability Evaluation of Long-Circulating and pH-Sensitive Liposomes Containing Ursolic Acid

**DOI:** 10.1155/2013/467147

**Published:** 2013-08-04

**Authors:** Sávia Caldeira de Araújo Lopes, Marcus Vinícius Melo Novais, Cláudia Salviano Teixeira, Kinulpe Honorato-Sampaio, Márcio Tadeu Pereira, Lucas Antônio Miranda Ferreira, Fernão Castro Braga, Mônica Cristina Oliveira

**Affiliations:** ^1^Departamento de Produtos Farmacêuticos, Faculdade de Farmácia, Universidade Federal de Minas Gerais, Avenida Antônio Carlos, 6627, 31270-901 Belo Horizonte, MG, Brazil; ^2^Centro de Microscopia, Universidade Federal de Minas Gerais, Avenida Antônio Carlos, 6627, 31270-901 Belo Horizonte, MG, Brazil; ^3^Centro de Desenvolvimento de Tecnologia Nuclear (CDTN)/Comissão Nacional de Energia Nuclear (CNEN), Avenida Antônio Carlos, 6627, 31270-901 Belo Horizonte, MG, Brazil

## Abstract

Cancer is one of the leading causes of death worldwide. Although several drugs are used clinically, some tumors either do not respond or are resistant to the existing pharmacotherapy, thus justifying the search for new drugs. Ursolic acid (UA) is a triterpene found in different plant species that has been shown to possess significant antitumor activity. However, UA presents a low solubility in aqueous medium, which presents a barrier to its biological applications. In this context, the use of liposomes presents a promising strategy to deliver UA and allow for its intravenous administration. In this work, long-circulating and pH-sensitive liposomes containing UA (SpHL-UA) were developed, and their chemical and physicochemical properties were evaluated. SpHL-UA presented adequate properties, including a mean diameter of 191.1 ± 6.4 nm, a zeta potential of 1.2 ± 1.4 mV, and a UA entrapment of 0.77 ± 0.01 mg/mL. Moreover, this formulation showed a good stability after having been stored for 2 months at 4°C. The viability studies on breast (MDA-MB-231) and prostate (LNCaP) cancer cell lines demonstrated that SpHL-UA treatment significantly inhibited cancer cell proliferation. Therefore, the results of the present work suggest the applicability of SpHL-UA as a new and promising anticancer formulation.

## 1. Introduction

 Ursolic acid (UA) is a triterpenoid compound that exists abundantly in the plant kingdom. UA has been reported to have an interesting bioactivity, including anti-inflammatory [1, 2], antihyperlipidemic [3, 4], and hepatoprotective [3] properties. Recent studies have shown that UA has revealed antitumor effects and cytotoxic activity towards various types of cancer cell lines [5–12].

 However, although UA presents the advantage of low toxicity, the clinical application of UA is limited due to several problems, such as its limited water solubility, which leads to a low bioavailability and poor pharmacokinetics *in vivo* and subsequently restricts its effectiveness [13]. Another limitation is its nonspecific distribution throughout the body when administered intravenously. Thus, it is desirable to explore novel formulations of UA that overcome these inconveniences [12]. In this context, the use of nanosystems as carriers, such as liposomes, consists of a promising strategy to deliver this substance and allow for its intravenous administration. Moreover, considering the antitumor action of this triterpene, the use of nanocarriers as vehicles can enable the targeting of this compound to the tumor region, providing greater therapeutic efficacy. 

 Liposomes are well-recognized drug delivery systems which can act as biocompatible, biodegradable, and nonimmunogenic drug carriers [14]. A major drawback of conventional liposomes is the rapid uptake of these particles *in vivo* by cells of the mononuclear phagocyte system (MPS) [15, 16]. Several different strategies have been developed to overcome these difficulties, such as coating the surface of the liposomes with inert molecules, like polyethylene glycols (PEG), which form a spatial barrier. The presence of PEG on the surface of the liposomal carrier has proven to extend blood-circulation time while reducing MPS uptake (stealth liposomes). This technology has resulted in a large number of nanocarriers encapsulating active molecules, with high target efficiency and activity [17–19].

 In spite of the strategies mentioned previously, conventional and long-circulating liposomes may present a slow release of the active substance or may be unable to fuse with the endosome after internalization. As such, polymorphic liposomes have been developed to overcome these problems, mainly due to the fact that these liposomes become reactive when submitted to membrane changes triggered by pH, variations in temperature, or surface charge alterations. A pH-sensitive liposome is generally stable at physiological pH but can undergo destabilization and acquire fusogenic properties under acid conditions, thus leading to the release of its aqueous contents [20–22]. The development of this kind of liposome was proposed after the observation that some pathological tissues, including tumors or areas of inflammation and infection, as compared to normal tissues, reveal an acid environment [23]. Furthermore, the endosome formed during the cellular internalization of liposomes also presents an acidic pH which favors their fusion and release of entrapped drugs [21]. 

Therefore, in the present study, long-circulating and pH-sensitive liposomes consisting of dioleoylphosphatidylethanolamine (DOPE), cholesteryl hemisuccinate (CHEMS), and distearoylphosphatidylethanolamine-polyethyleneglycol_2000_ (DSPE-PEG_2000_) containing UA (SpHL-UA) were developed and their chemical and physicochemical properties were evaluated. In addition, the effect of SpHL-UA towards cancer cell lines viability, such as MDA-MB-231 and LNCaP, was also investigated.

## 2. Materials and Methods

### 2.1. Materials

 DOPE and DSPE-PEG_2000_ were supplied by Lipoid GmbH (Ludwigshafen, Germany). UA, CHEMS, phosphate saline buffer, sodium hydroxide, Triton X-100, sodium dodecyl sulfate (SDS), MTT reagent (3-(4, 5-dimethylthiazolyl-2)-2,5-diphenyltetrazolium bromide), fetal bovine serum (FBS), penicillin, and streptomycin were obtained from Sigma Chemical Company (St. Louis, MO, USA). Roswell Park Memorial Institute Medium 1640 (RPMI) and Dulbecco's Modified Eagle's Medium (DMEM) were purchased from Gibco (Grand Island, NE, USA). Methanol was obtained from Fisher Scientific (NJ, USA). All other chemicals used in this study were of analytical grade. 

The cancer cell line MDA-MB-231 (human breast adenocarcinoma) was kindly supplied by Professor Dr. Alfredo Miranda de Goes (Laboratory of Biochemistry and Immunology, Institute of Biological Sciences, Universidade Federal de Minas Gerais, Belo Horizonte, Brazil). The LNCaP (human prostate carcinoma) cancer cell was purchased from American Type Culture Collection (ATCC) (Manassas, VA, USA). 

### 2.2. Preparation of SpHL-UA

 For SpHL-UA preparation, the present study employed the lipid hydration method described by Bangham et al. [24]. Briefly, 11.4 mL of DOPE 50 mM, 7.6 mL of CHEMS 50 mM, and 5 mL of DSPE-PEG_2000_ 10 mM, dissolved in chloroform, were transferred to a round bottom flask amounting a total lipid concentration of 20 mM (molar ratio of 5.7 : 3.8 : 0.5, resp.). Similarly, 22.8 mL of DOPE 50 mM, 15.2 mL of CHEMS 50 mM, and 10 mL of DSPE-PEG_2000_ 10 mM, dissolved in chloroform, in a total lipid concentration of 40 mM (molar ratio of 5.7 : 3.8 : 0.5, resp.) were also transferred to a round bottom flask. UA equivalent at 0.1% (w/v) or 0.05% (w/v) was added to the lipid solution. A lipid film was obtained by evaporating the chloroform under reduced pressure. Next, the lipid film was hydrated with an amount of 3.8 mL of NaOH 0.1 M, for 20 mM liposomes, and 7.6 mL of NaOH 0.1 M for 40 mM liposomes to promote the complete ionization of CHEMS. Finally, phosphate buffersaline (PBS), pH 7.4, was added in amount of 46.2 mL for 20 mM liposomes and 42.4 mL for 40 mM liposomes. The obtained mixture was subjected to vigorous shaking in a vortex, producing multilamellar liposomes. The resulting multilamellar vesicles were calibrated using a single-stage high pressure homogenizer, model APV 2000 (APV, Albertslund, Denmark) in recirculation mode. The pressure of the homogenizer was adjusted to 500 bar. The minimum volume of processed samples was 110 mL, and all homogenizations were carried out at room temperature. Each cycle was equal to the passage of the total volume of the sample through the homogenization chamber in a total of twelve cycles. Nonentrapped UA was separated by ultrafiltration, using a polyethersulfone membrane (Millipore Pellicon XL device; Biomax, cut off 500 kDa; MA, USA) connected to a tangential flow filtration system (Labscale; Millipore, MA, USA). The liposome suspension (100 mL) was transferred to an initial container and pumped to the filtration membrane. Nonentrapped UA (permeated) was recovered in a flask, while purified SpHL-UA was returned to the initial container. This process was maintained until a concentrated SpHL-UA suspension reached the final volume of 25 mL. After the cycle of ultrafiltration was completed, the permeated was taken and the nonentrapped UA concentration was measured by high performance liquid chromatography (HPLC). In addition, the purified SpHL-UA was diluted with PBS buffer pH 7.4 to the final volume of 50 mL. Next, the evaluation of drug entrapment was performed by HPLC (see Section 2.3.1).

 In order to prepare sterile SpHL-UA, samples were irradiated at 10 kGy, for about 30 minutes (dose rate of 20.57 kGy/h), using a multipurpose panoramic irradiator equipped with a Cobalt-60 source (IR-214 model, MDS Nordion, Canada) at the Center for the Development of Nuclear Technology-National Commission on Nuclear Energy (CDTN-CNEN, Brazil).

### 2.3. SpHL-UA Characterization

#### 2.3.1. Drug Entrapment Determination

 The evaluation of drug entrapment was performed after having purified the SpHL-UA, which was solubilized by adding methanol. The total amount of UA in the liposomes was also measured after SpHL-UA had been dissolved in methanol, before purification procedures by ultrafiltration. After preparing the samples, as mentioned previously, UA quantification was performed by HPLC. The chromatographic apparatus consisted of a model 515 pump, a model 717 Plus autoinjector, and a model 2487 variable wavelength UV detector (Waters Instruments, USA) controlled by Empower software. Separations were performed using a 25 cm × 4 mm, 5 *μ*m LiChrosorb, RP-18 column (Merck SA, Germany). The eluent system consisted of a 1 : 1 methanol/water mixture, and the flow rate was 1.5 mL·min^−1^. Samples (20 *μ*L) were injected, and the absorbance of the eluate was monitored at 210 nm. 

 The specificity of the HPLC method was carried out by comparing the peak retention time of UA and the peaks obtained after having injected liposomes without UA (blank liposomes). The linear response was evaluated in the concentration range of 5–60 *μ*g/mL of UA. The precision of the method was assessed considering repeatability and intermediate precision at three concentration levels of UA (5, 20, and 60 *μ*g/mL) on three different days. 

#### 2.3.2. Measurements of Size and Zeta Potential

 The mean diameter of SpHL-UA was determined by photon correlation spectroscopy (PCS) at 25°C and at an angle of 90°. The zeta potential was evaluated by determining the electrophoretic mobility at an angle of 90°. The measurements were performed using the Zetasizer 3000 HAS (Malvern Instruments Ltd, Worcestershire, UK). The samples were diluted with PBS buffer solution.

#### 2.3.3. Transmission Electron Microscopy (TEM)

 Morphological examination of SpHL-UA was performed by means of TEM using a negative staining method. The liposomes were placed on a formvar-coated copper grid and stained with a 2% (w/v) phosphotungstic acid solution containing 0.5% (w/v) bovine serum albumin and 0.5% (w/v) saccharose. The stained samples were characterized using a Tecnai G2 12 Spirit Biotwin FEI at 80 kV (Centro de Microscopia, Universidade Federal de Minas Gerais, Belo Horizonte, Brazil).

#### 2.3.4. Stability Study

 The determination of the storage stability of SpHL-UA was performed at 15, 30, and 60 days after preparation. These formulations were maintained at 4°C. The parameters evaluated included mean diameter, zeta potential, and drug entrapment. The mean values of these parameters were compared with those obtained at time zero. 

### 2.4. *In Vitro* Studies

#### 2.4.1. Cell Cultures

 The effect of SpHL-UA on cell viability was evaluated on cancer cell lines MDA-MB-231 and LNCaP. Cells were cultured in RPMI 1640 for LNCaP cells and DMEM medium for MDA-MB-231; all media were supplemented with 10% (v/v) FBS and antibiotics (100 *μ*g/mL streptomycin and 100 UI/mL penicillin). All cultures were kept in a humidified incubator with 5% CO_2_ at 37°C.

#### 2.4.2. Analysis of Cell Viability

 Cell proliferation was measured by MTT assay based on the reduction of tetrazolium salt to formazan crystals by living cells [25]. Briefly, aliquots containing 1 × 10^4^ (LNCaP) and 2 × 10^3^ (MDA-MB-231) cells/well were seeded into 96-well plates. After 24 h of incubation at 37°C and 5% CO_2_, freshly prepared solutions of free UA and SpHL-UA were added to the wells. The concentrations assayed were 1.2, 2.5, 5, 10, 20, and 40 *μ*M of UA. Free UA was dissolved in DMSO prior to dilution. After 48 h of incubation at 37°C and 5% CO_2_, the medium with treatment was removed and discarded. Subsequently, 100 *μ*L of culture medium containing 10% (v/v) FBS and 0.5 mg of tetrazolium/mL was added to each well of the culture plate. After two hours of incubation, MTT crystals were solubilized in 100 *μ*L of a solution containing 10% (w/v) SDS in 0.01 M HCl. Cell viability was estimated by measuring the rate of mitochondrial reduction of MTT determined by evaluating the absorbance of the converted dye at a wavelength of 595 nm. Absorbance values of the wells in which the cells were maintained in medium alone were considered to be 100% of cell viability. The control groups included treatment with DMSO and blank liposomes. Data were expressed as percentage of cell viability compared to the control (mean ± SD). The IC_50_ values (i.e., UA concentration resulting in 50% of carcinoma cells viability) of free UA and SpHL-UA were calculated using Graphpad Prism 5.0 (Graphpad Software Inc., San Diego, USA). At least three independent experiments were performed for each evaluated cancer cell line.

## 3. Statistical Analysis 

 Data were subjected to statistical analysis using the one-way analysis of variance (ANOVA), followed by the Bonferroni test, and *P* values of less than 0.05 were regarded as significant (Graphpad Prism 5.0, Graphpad Software Inc., San Diego, USA). The results were expressed as mean values ± standard deviation (S.D.). 

## 4. Results and Discussion

### 4.1. HPLC Method Validation

 The HPLC method showed adequate specificity. No interference of the liposome components could be identified, since no overlaps of peaks were detected after the injection of UA at the set wavelengths (data not shown). The linear response was obtained in the evaluated UA concentration range (5–60 *μ*g/mL) with a correlation coefficient greater than 0.999 and a linear equation of *y* = 5001*x* + 2098. The precision of the method was also confirmed. Whatever the UA concentration level, the overall results showed relative standard deviation values of lower than 5% in all experiments (data not shown). These results were in agreement with requirements for analytical assays [26].

### 4.2. SpHL-UA Characterization

 The behavior of liposomes in storage conditions and biological mediums is determined by factors such as the size and surface charge of vesicles and the quantity of entrapped solute. Thus, it is of utmost importance to have as much information as possible regarding these parameters to ensure the efficacy and stabilization of the liposome formulation [27].

 The chemical and physicochemical properties of SpHL-UA, prepared with different lipid and drug concentrations, are summarized in Table 1.

 Analyzing Table 1, it could be observed that the SpHL 20UA1 formulation mostly called attention due to its high concentration of UA embedded in the lowest level of lipid concentration (20 mM). In addition, we can observe that the presence of UA leads to an increase in size of vesicles for all formulations. Moreover, by analyzing the distribution of vesicles of SpHL 20UA1, it could be observed that approximately 88% of the vesicles had to be smaller than 300 nm (Table 2). It is well known that liposomal preparations for anticancer treatment must present small-sized vesicles to comply with safety requirements and improve therapeutic efficacy [28]. Liposome size is extremely relevant to deliver anticancer agents to tumor tissue because these particles are known to accumulate in the tumor area due to the leaky vasculature-enhanced permeability and retention (EPR) effect. This effect occurs due to the anatomic differences between normal and cancerous tissue because capillaries in the tumor area possess increased permeability. This defective vascular architecture coupled with poor lymphatic drainage induces an enhanced permeability and retention. Therefore, liposomes in the range of 100 to 150 nm have been shown to preferentially accumulate in tumors due to this EPR effect. Large pores may exist in the tumor vessel wall that allow the penetration of liposomes up to the size of 400 nm in diameter [29–31]. Moreover, it is important to note that the size range is a compromise between loading efficiency of liposomes (increases with increasing size), liposome stability (decreases with increasing size above an optimal 80–200 nm range), and ability to extravasate (decreases with increasing size) [32]. Thus, the size of SpHL 20UA1 may contribute to their therapeutic success.

 Therefore, SpHL 20UA1 was selected for subsequent studies. First, it was subjected to a stability study over a 60-day period. It is worth noting that SpHL 20UA1 showed a good stability in terms of mean vesicle size, zeta potential, and UA entrapment after storage for 2 months at 4°C (Table 3). 

 A typical phenomenon of instability in the liposome formulation is the increase in particle size due to the aggregation or the fusion of unstable liposomes upon storage. An increase in particle size of liposomes generally results in a rapid uptake by MPS with a subsequent rapid clearance and a short half-life. Moreover, the fusion of vesicles leads to the leakage of the encapsulated drug. Thus, controlling and maintaining liposomes at small and uniform sizes are critical in developing a viable pharmaceutical product [33].

 Zeta potential is an other important parameter to the stability of colloidal formulations. Generally, zeta potential values of 30 mV (absolute value) and above characterize a stable formulation, since the aggregation of the particles is less likely to occur due to electrical repulsion forces [27]. However, in this work, as shown by zeta potential measurements, the liposome surface charges were near neutrality, but this seems not to have affected its stability (Table 3). The maintenance of vesicle diameter over time may well be due to the presence of PEG chains on the liposome surface, which could prevent vesicle aggregation, improving the stability of the formulations [18]. This fact can be explained by the steric repulsion provoked by the polymer chains of PEG_2000_-lipids [34].

 Stability in terms of UA entrapment can be explained by the UA lipophilic character (log⁡*P* = 7.92) [35] which could enable a stronger interaction with phospholipids in a liposome bilayer, in turn preventing drug release during storage.

#### 4.2.1. TEM Measurements

**Figure 1:**
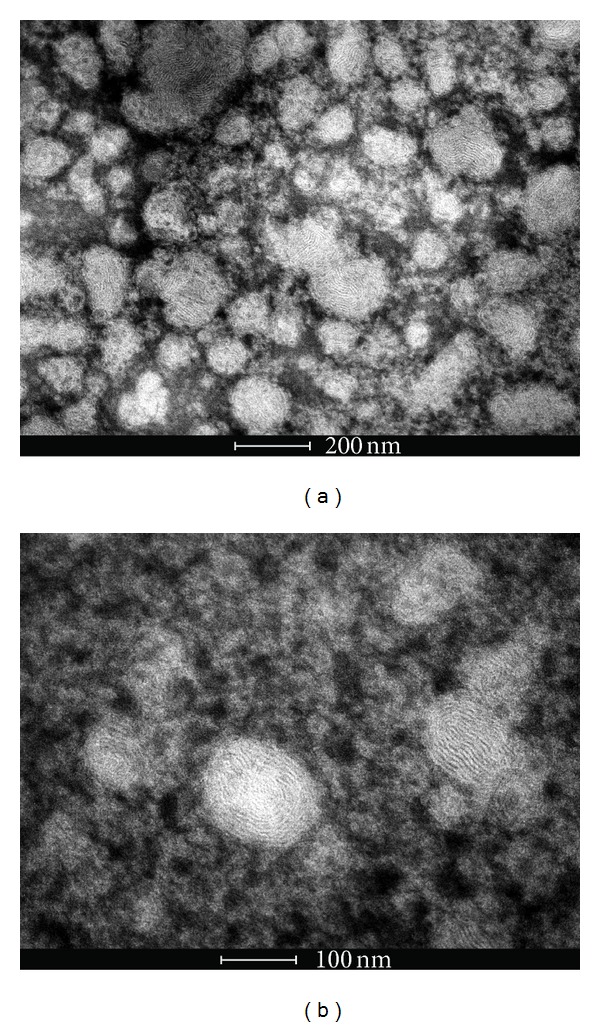
TEM photomicrographs obtained for SpHL 20UA1 in different fields ((a) and (b)).

 The images of SpHL 20UA1 obtained by TEM (Figure 1) allowed for the viewing of multilamellar vesicles of varying diameters, predominantly of vesicle sizes of less than 100 nm, which were consistent with the results obtained from the particle sizes measured by PCS technique (shown in Table 2).

### 4.3. Cell Viability Assay

 To evaluate the effects of SpHL 20UA1 on breast and prostate cancer cell lines viability, an MTT assay was employed. The IC_50_ values were calculated for each cell line and are summarized in Table 4. It is worth noting that effects on cell viability after control groups treatment (DMSO and blank liposomes) were negligible for both cancer cell lines.

 In Table 4, it can be observed that IC_50_ value obtained after SpHL 20UA1 treatment (48 h) was significantly lower than IC_50_ value obtained after UA free treatment (*P* < 0.05) for MDA-MB-231 cancer cell line. This result indicates that SpHL can improve UA delivery in this breast cancer cell line. There was not a significant difference among the IC_50_ values obtained after SpHL 20UA1 and free UA treatments (*P* > 0.05) for LNCaP cancer cell line. These findings revealed that the incorporation of UA into SpHL did not provoke any impairment in the effect of UA on carcinoma cells viability. Thus, the use of SpHL 20UA1 may be a promising strategy to carry UA and allow for its intravenous administration. 

## 5. Conclusion

 In conclusion, the results of the present study demonstrated that SpHL-UA had a good stability in terms of mean vesicle size, zeta potential, and UA entrapment after storage for 2 months at 4°C. Thus, the production of these liposomes proposed in this work led to liposomal dispersion with features suitable for future *in vivo* applications. This work also investigated the effects of SpHL-UA on breast and prostate cancer cell lines viability, which demonstrated that, after 48 h, this treatment significantly inhibited cancer cell proliferation, as shown by MTT assay. Results from this study indicate that SpHL-UA can be an interesting delivery system for the pharmaceutical formulation of UA and may represent a good and useful chemotherapy agent for breast and prostate cancer treatment. Further evaluations, such as pharmacokinetics studies and antitumor activity *in vivo*, should be performed to confirm these expectations.

## Figures and Tables

**Table 1:** Chemical and physicochemical properties of liposomes containing or not UA^a^.

Formulation	Mean diameter (nm)	Zeta potential (mV)	UA entrapment (mg/mL)
SpHL 20B^b^	105.8 ± 3.3	3.2 ± 1.0	—
SpHL 20UA1^c,h^	191.1 ± 6.4	1.2 ± 1.4	0.77 ± 0.01
SpHL 20UA0.5^d,h^	149.4 ± 1.5	1.7 ± 2.6	0.38 ± 0.01
SpHL 40B^e^	107.3 ± 2.6	2.9 ± 0.4	—
SpHL 40UA1^f,h^	140.4 ± 1.2	4.1 ± 1.3	0.78 ± 0.05
SpHL 40UA0.5^g,h^	122.8 ± 3.1	4.4 ± 0.5	0.35 ± 0.04

^a^Values are expressed as mean ± S.D. (*n* = 3). ^ b^Lipid concentration is equal to 20 mM, without UA. ^c^Lipid concentration is equal to 20 mM, and UA concentration is of 1 mg/mL. ^d^Lipid concentration is equal to 20 mM, and UA concentration is of 0.5 mg/mL. ^e^Lipid concentration is equal to 40 mM, without UA. ^f^Lipid concentration is equal to 40 mM, and UA concentration is of 1 mg/mL. ^g^Lipid concentration is equal to 40 mM, and UA concentration is of 0.5 mg/mL. ^h^There is a significant difference among the mean diameter values of SpHL samples containing UA and the respective SpHL samples without UA at the same lipid concentration (*P *< 0.05).

**Table 2:** Distribution of vesicle diameter (%) for SpHL 20UA1^a^.

Mean diameter (nm)	Distribution of vesicle diameter (%)			
	≤100 nm	≤300 nm	300–500 nm	≥500 nm
191.1 ± 6.4	72.4 ± 14.7	88.4 ± 7.3	7.1 ± 4.7	4.4 ± 2.6

^a^Each value represents the mean ± S.D (*n* = 3).

**Table 3:** Physicochemical and chemical stability of SpHL 20UA1^a^.

	Mean diameter (nm)	Zeta potential (mV)	UA entrapment (mg/mL)
Time 0	191.1 ± 6.4^b^	1.2 ± 1.4^b^	0.77 ± 0.01^b^
15 days	210.3 ± 15.2^b^	−1.7 ± 3.1^b^	0.76 ± 0.01^b^
30 days	221.9 ± 25.3^b^	−1.6 ± 3.5^b^	0.76 ± 0.01^b^
60 days	212.7 ± 15.3^b^	−1.4 ± 3.6^b^	0.77 ± 0.01^b^

^a^Each value represents the mean ± S.D (*n* = 3).^ b^There is no significant difference among the means indicated at the same column (*P *> 0.05). All means were compared with time zero.

**Table 4:** IC_50_ values for cancer cell lines^a^.

Cell lines	IC_50 _(*µ*M) Free UA	IC_50 _(*µ*M) SpHL 20UA1
MDA-MB-231	13.07 ± 1.54^b^	8.13 ± 2.3
LNCaP	2.49 ± 0.53^c^	2.68 ± 1.06

^a^Each value represents the mean ± S.D (*n* = 3). ^b^There is a significant difference among the means indicated at the same line (*P *< 0.05). ^c^There is no significant difference among the means indicated at the same line (*P *> 0.05).
